# In Vivo Efficacy of *Lacticaseibacillus rhamnosus* L8020 in a Mouse Model of Oral Candidiasis

**DOI:** 10.3390/jof7050322

**Published:** 2021-04-21

**Authors:** Rei Ito, Yuichi Mine, Yoshie Yumisashi, Reina Yoshioka, Misa Hamaoka, Tsuyoshi Taji, Takeshi Murayama, Hiroki Nikawa

**Affiliations:** 1 Department of Oral Biology & Engineering, Division of Oral Health Sciences, Graduate School of Biomedical and Health Sciences, Hiroshima University, Hiroshima 734-8553, Japan; m192786@hiroshima-u.ac.jp (R.I.); m200583@hiroshima-u.ac.jp (Y.Y.); m201787@hiroshima-u.ac.jp (M.H.); taji@hiroshima-u.ac.jp (T.T.); hirocky@hiroshima-u.ac.jp (H.N.); 2 Department of Medical System Engineering, Division of Oral Health Sciences, Graduate School of Biomedical and Health Sciences, Hiroshima University, Hiroshima 734-8553, Japan; m203086@hiroshima-u.ac.jp (R.Y.); murayatk@hiroshima-u.ac.jp (T.M.)

**Keywords:** oral candidiasis, *Candida albicans*, probiotics, *Lacticaseibacillus rhamnosus* L8020

## Abstract

Oral candidiasis presents with multiple clinical manifestations. Among known pathogenic *Candida* species, *Candida albicans* is the most virulent and acts as the main causative fungus of oral candidiasis. Novel treatment modalities are needed because of emergent drug resistance and frequent candidiasis recurrence. Here, we evaluated the ability of *Lacticaseibacillus rhamnosus* L8020, isolated from healthy and caries-free volunteers, to prevent against the onset of oral candidiasis in a mouse model. Mice were infected with *C. albicans*, in the presence or absence of *L. rhamnosus* L8020. The mice were treated with antibiotics and corticosteroid to disrupt the oral microbiota and induce immunosuppression. We demonstrated that oral consumption of *L. rhamnosus* L8020 by *C. albicans*-infected mice abolished the pseudomembranous region of the mouse tongue; it also suppressed changes in the expression levels of pattern recognition receptor and chemokine genes. Our results suggest that *L. rhamnosus* L8020 has protective or therapeutic potential against oral candidiasis, which supports the potential use of this probiotic strain for oral health management.

## 1. Introduction

Oral candidiasis presents with various clinical manifestations, including the classic white thrush lesion that develops into pseudomembranes resembling milk curds [1]. *Candida albicans* is the most virulent known pathogenic *Candida* species and the most common causative fungus of oral candidiasis. Importantly, *C. albicans* is an opportunistic fungal pathogen of humans, which colonizes the skin and mucosal surfaces of most healthy people in an asymptomatic manner [2]. However, changes in host immunity (e.g., T-cell deficiency) may not control the colonization of *C. albicans* on mucosal surfaces, thereby resulting in the development of disease [3]. The detection rates of *C. albicans* in patients with oral candidiasis are 15–71% in denture wearers and 80–95% in human immunodeficiency virus-infected individuals [4]. The core pathogenic mechanism of *C. albicans* involves its ability to switch between yeast and hyphal morphologies; *C. albicans* attached to host surfaces can switch to an invasive hyphal and filamentous morphology, facilitating invasion of the epithelium [5].

Probiotics have been defined as “Live microorganisms which when administered in adequate amounts confer a health benefit on the host” by the Food and Agriculture Organization/World Health Organization [6]; this definition has been supported by the International Scientific Association for Probiotics and Prebiotics [7]. The use of probiotics has been investigated for the prevention of health problems, including digestive disorders, allergic disorders, and dental and oral diseases [8,9].

*Lactobacillus* spp. have a long history of safe usage as probiotics, and over 200 species have been classified within the *Lactobacillus* genus [10]. Some *Lactobacillus* spp. (e.g., *L. rhamnosus* and *L. reuteri*) can diminish the influence of *C. albicans* through the release of anti-fungal molecules and/or regulation of the immune system [11,12]. Recently, the genus *Lactobacillus* was proposed to undergo reclassification into 25 genera, on the basis of whole genome sequence analyses [13]. Following this reclassification, lactobacilli were grouped into robust clades that share ecological and metabolic characteristics.

We previously isolated 42 *Lactobacillus* spp. from healthy volunteers who had no caries or caries-treatment experience. Among the 42 isolates, *L. rhamnosus* L8020 (reclassified as *Lacticaseibacillus rhamnosus* on the basis of [13]) showed >95% growth inhibition of *Porphyromonas gingivalis*, *Streptococcus mutans*, *Streptococcus sobrinus*, and *C. albicans* in vitro [8]. Moreover, the oral isolate *L. rhamnosus* L8020 may help to maintain mucosal homeostasis through the induction of transient epithelial cell activation [14]. These properties suggested that *L. rhamnosus* L8020 may be a promising probiotic strain for oral health management. Additionally, our clinical studies revealed that *L. rhamnosus* L8020 could reduce the oral burden of mutans streptococci and the risk of periodontal disease [8,15].

In this study, we investigated the efficacy of *L. rhamnosus* L8020 for preventing oral candidiasis in vivo in a mouse model of *C. albicans* infection. ICR mice, a typical outbred stock, have more variability in genetic characteristics and faster growth rate than inbred mice; disease models using ICR mice are widely used in studies for probiotics [4,16,17,18]. The ICR mice were treated with antibiotics and corticosteroid to disrupt the oral microbiota and induce immunosuppression [17,18]. We examined clinical symptoms of pseudomembranous candidiasis in the mice, then analyzed the local expression trends of pattern recognition receptors (PRRs) and chemokines during the early stages of *C. albicans* infection.

## 2. Materials and Methods

### 2.1. Bacterial Strains

*L. rhamnosus* L8020, previously isolated from healthy and caries-free volunteers (clinical isolates, Hiroshima University Hospital, Hiroshima, Japan) [8], was used as the probiotic strain in this study. *C. albicans* GDH18 (ATCC MYA-274), an oral isolate obtained from the routine microbiology services of the Glasgow Dental Hospital and School (Glasgow, UK), was used for infection in this study. *L. rhamnosus* L8020 was preincubated in *Lactobacillus* de Man, Rogosa and Sharpe broth (Difco, Tokyo, Japan) for 24 h at 37 °C. *C. albicans* GDH18 was cultured aerobically in Sabouraud dextrose broth (Japan Becton Dickinson Company, Tokyo, Japan) at 37 °C [19].

### 2.2. Animals

Animal care and experiments were performed in accordance with the guidelines of institutional authorities and approved by the Ethics Committee for Animal Experiments at Hiroshima University (Approval No. A20-57). Six-week-old female ICR mice were purchased from Charles River Laboratories Japan (Yokohama, Japan). The mice were acclimatized to the experimental facility for 1 week to aid in stress relief. Twenty-eight mice were randomly divided into four groups of seven animals each: no *C. albicans* GDH18 infection and drinking water (control group); no *C. albicans* GDH18 infection and *L. rhamnosus* L8020 consumption (L8020 group); *C. albicans* GDH18 infection and drinking water (*C. a* GDH18 group); and *C. albicans* GDH18 infection with *L. rhamnosus* L8020 consumption (*C. a* GDH18 + L8020 group).

### 2.3. In Vivo Oral Candidiasis Model

**Figure 1:**
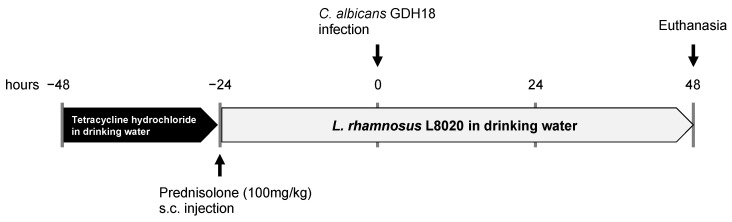
Schematic of oral candidiasis establishment and treatment in a mouse model.

To induce oral candidiasis in the mice, we used a previously described protocol [17,18], with minor modifications. Mice were administered 5 mg/mL tetracycline hydrochloride (AdipoGen, San Diego, CA, USA) via drinking water, beginning at 48 h before *C. albicans* GDH18 infection; they then received 100 mg/kg prednisolone (Kyoritsu Seiyaku Corporation, Tokyo, Japan) subcutaneously, beginning at 24 h before *C. albicans* GDH18 infection. Beginning at 24 h before infection, mice were also freely administered 1.0 × 10^6^ cells/mL of *L. rhamnosus* L8020 via drinking water; this was continued until the end of the experimental period. Mice were anesthetized using a combination of 0.3 mg/kg medetomidine, 4 mg/kg midazolam, and 5 mg/kg butorphanol; they were then orally infected with 1.0 × 10^9^ cells/mL of *C. albicans* GDH18 by means of rubbing *C. albicans*-impregnated cotton swabs inside all parts of the mouth, followed by *C. albicans*-impregnated cotton swab placement on the dorsum of tongue for 90 min. The mice were euthanized at 48 h after *C. albicans* infection (total experimental duration of 96 h; Figure 1). To assess the severity of disease, tongue regions with pseudomembranous candidiasis were measured on digital images using ImageJ software (version 1.53, National Institutes of Health, Bethesda, MD, USA). The region of interest was defined as 10 cm from the tongue tip; all measurements were performed in that region of the tongue.

### 2.4. Histological Assessments

Mouse whole tongues were harvested and fixed in 10% formaldehyde neutral buffer solution at 4 °C, then embedded in paraffin. Samples were cut in 12 μm-thick serial cross sections, then stained using periodic acid–Schiff and hematoxylin methods. Digital photographs of all histological slides were captured using a BZ-X710 microscope (KEYENCE, Osaka, Japan)

### 2.5. Real-Time Quantitative RT-PCR Analysis

Total RNA was isolated from palatal gingival tissues using TRIzol reagent (Invitrogen, Carlsbad, CA, USA). First-strand cDNA was synthesized from 100 ng of total RNA using ReverTra Ace qPCR RT Master Mix (Toyobo, Osaka, Japan). cDNA samples were mixed with Rotor-Gene Probe PCR Master Mix (Qiagen, Tokyo, Japan) for real-time quantitative reverse transcriptase polymerase chain reaction analyses; the analyses were performed using a Rotor-Gene 6000 (Qiagen). β-actin was used as an internal control to correct for amplification variability caused by differences in starting total RNA concentrations. The polymerase chain reaction primers and probes used in this study are shown in Supplementary Table S1.

### 2.6. Statistical Analysis

Statistical analyses were performed using IBM SPSS Statistics software, version 27.0 (IBM Corp., Armonk, NY, USA). One-way analysis of variance and Tukey’s multiple comparison test were used to analyze differences among groups; *p*-values < 0.05 were considered statistically significant.

## 3. Results

### 3.1. Anti-Fungal Efficacy of L. rhamnosus L8020 in a Mouse Model of Oral Candidiasis

**Figure 2:**
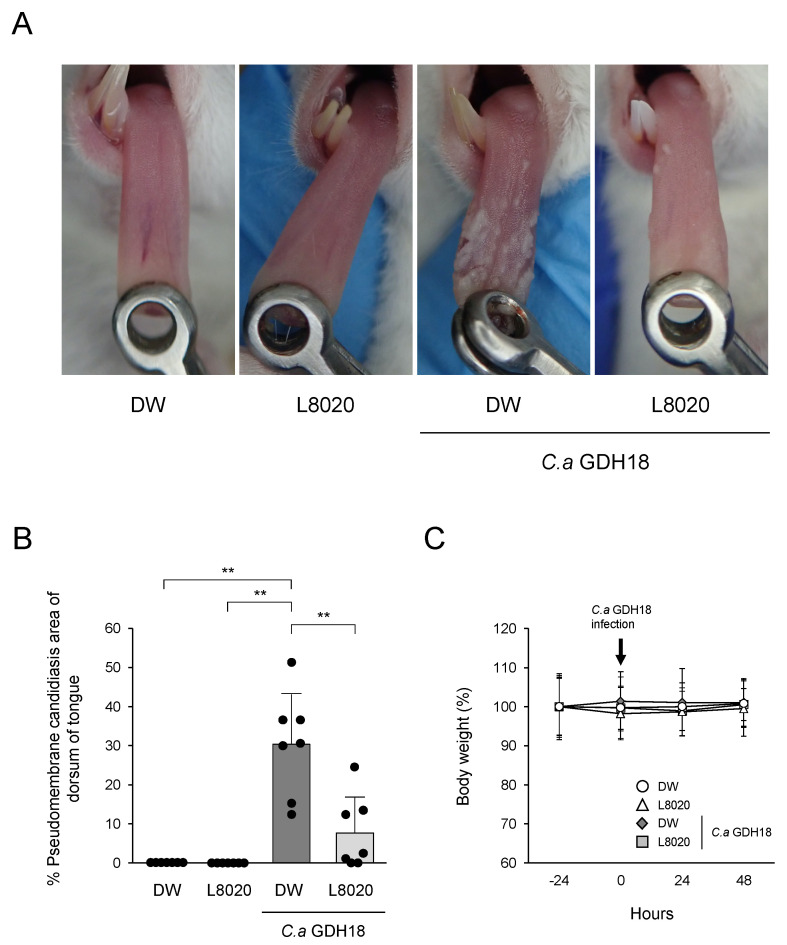
Treatment with *L. rhamnosus* L8020 reduced the pseudomembranous candidiasis region of the tongue. (**A**) Macroscopic features on the tongue at 48 h following inoculation with *C. albicans* GDH18. Fungal lesions on tongues of representative mice in each group are shown. (**B**) Quantification of the pseudomembranous candidiasis region of the tongue (*n* = 7 mice per group). Data points indicate individual values from each animal. (**C**) Time course of weight change (%) in control (circles), L8020 (triangles), *C. albicans* GDH18 (diamonds), and *C. albicans* GDH18 + L8020 (squares) groups of mice. Data in all graphs represent the means ± standard deviations. Asterisks indicate statistically significant differences. ** *p* < 0.01 by analysis of variance. DW: drinking water, L8020: *L. rhamnosus* L8020, *C. a* GDH18: *C. albicans* GDH18.

Mice infected with *C. albicans* GDH18 showed clinical symptoms of oral candidiasis (Figure 2A). Among these infected mice, the tongues of mice that had been administered *L. rhamnosus* L8020 showed smaller lesions, compared with the tongues of mice that had been administered plain drinking water (Figure 2A). Quantitative comparison of the pseudomembranous candidiasis region of the tongue among groups is shown in Figure 2B. *C. albicans* GDH18 infection led to a large pseudomembranous candidiasis region on the tongue. Importantly, infected mice that had been administered *L. rhamnosus* L8020 exhibited a significantly smaller region of pseudomembranous candidiasis, compared with infected mice that had been administered plain drinking water. All mice showed no differences in body weight during the experimental period (Figure 2C).

### 3.2. Histological Assessment of the Tongue Dorsum

**Figure 3:**
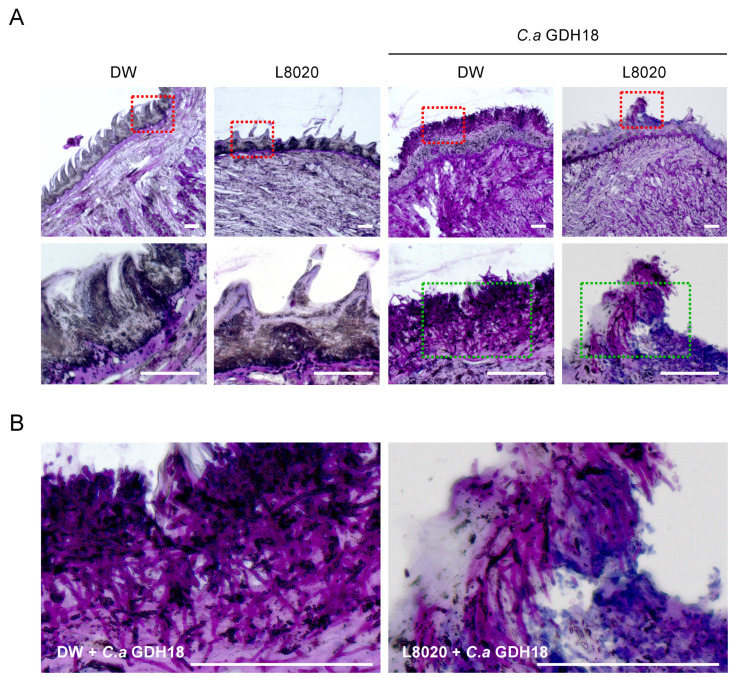
Histological assessments of paraffin-embedded sections of mouse tongues. (**A**) Histological evaluation of *C. albicans* GDH18 infection in mouse tongues. At 48 h after infection, sections of tongue were collected from the indicated mice, stained using the periodic acid–Schiff method, and visualized using a microscope. The first row was viewed at 4X magnification. The second row indicates the boxed region in the first row, viewed at 20X magnification. (**B**) Images of the green box region in panel A. Bars, 100 μm.

Histological analysis revealed that uninfected mice exhibited normal tongue tissues (Figure 3A), whereas mice with *C. albicans* GDH18 infection showed extensive fungal adhesions with hyphal invasion into the epithelial tissue (Figure 3A,B). In contrast, only a few of the mice treated with *L. rhamnosus* L8020 exhibited some sparsely colonized fungal cells within limited tongue regions (Figure 3A,B).

### 3.3. In Vivo Analyses of PRR and Chemokine Expression Levels

**Figure 4:**
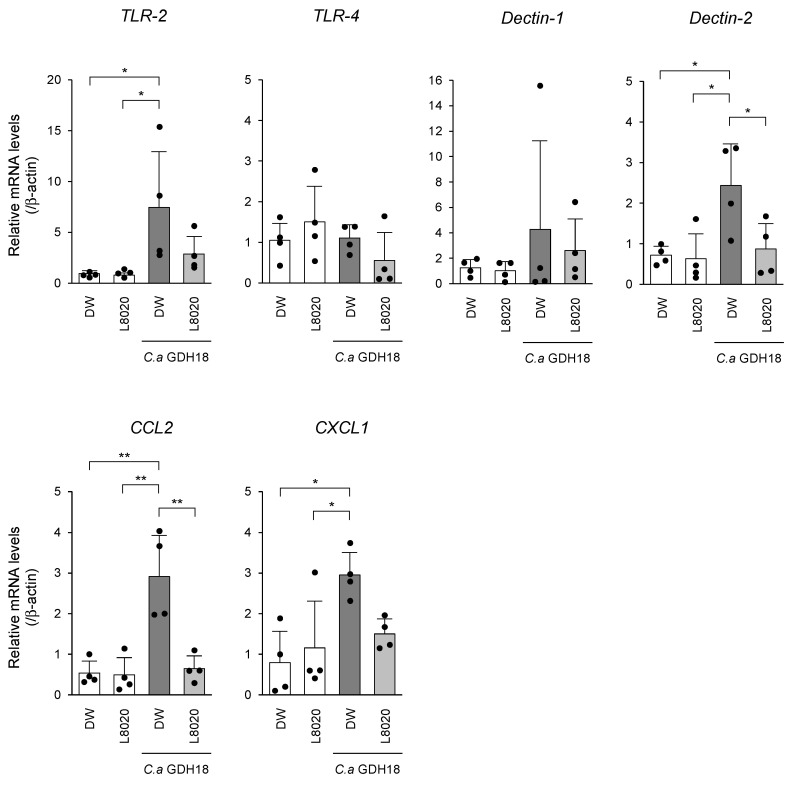
*C. albicans* GDH18-induced changes in PRR and chemokine genes expression levels were prevented and/or reduced by treatment with *L. rhamnosus* L8020 (*n* = 4 mice per group). Data points indicate individual values from each animal. Data in all graphs represent the means ± standard deviations. Asterisks indicate statistically significant differences. * *p* < 0.05, ** *p* < 0.01 by analysis of variance.

To examine whether *L. rhamnosus* L8020 affected the expression levels of *C. albicans* GDH18-induced PRRs and chemokines, Toll-like receptor (TLR) 2, TLR4, Dectin-1, Dectin-2, CCL2, and CXCL1/KC were analyzed in mouse palatal gingival tissues (Figure 4). *C. albicans* GDH18 infection led to enhanced mRNA expression levels of Dectin-2, TLR2, CCL2, and CXCL1/KC, compared with the levels in uninfected mice. *L. rhamnosus* L8020 treatment significantly reduced the expression levels of Dectin-2 and CCL2, and tended to reduce the expression levels of TLR2 and CXCL1/KC, following infection by *C. albicans* GDH18.

## 4. Discussion

This study demonstrated that treatment with the probiotic strain *L. rhamnosus* L8020 reduced the pseudomembranous candidiasis region of the tongue, while preventing and/or diminishing PRR and chemokine expression levels, in an immunosuppressed mouse model of oral candidiasis. The mechanisms of action of probiotics reportedly vary among species and strains [20]; however, these mechanisms are not fully established and new insights are emerging from genome engineering investigations [12]. Furthermore, animal model experiments and clinical studies provide important data regarding the efficacies of probiotics. Several animal models of oral, gastrointestinal and vaginal candidiasis have been proposed for analyses of fungal immunity and anti-fungal therapies [11,17,18,21,22,23,24,25,26,27]. These animal models have aided in elucidating molecular mechanisms related to candidiasis; they have also facilitated the evaluation of novel therapeutic approaches. Several *L. rhamnosus* strains (e.g., Lr-32, ATCC 7469, and CRL1332) have been reported to exhibit anti-fungal activity in vivo [11,22,27]. These reports supported the anti-fungal and anti-inflammatory potential of *L. rhamnosus*.

Colonization of the mucosal surface is an important anti-infection probiotic mechanism [28]. We previously revealed that *L. rhamnosus* L8020 demonstrates good adhesion to gingival epithelial-like cells; this adhesion ability is superior to the adhesion demonstrated by *Lactobacillus* spp. isolated from other organs and environments [14]. Thus, *L. rhamnosus* L8020 is expected to have a beneficial effect in protecting against *C. albicans* infection through the colonization of mucosal surfaces, which prevents expansion of the pseudomembranous candidiasis region on the tongue.

The immunological response to oral candidiasis is orchestrated by oral epithelial cells and their secreted proteins, as well as phagocytic cells and components of the adaptive immune system. Oral epithelial cells sense the early stages of an infection and activate the p-MKP1/c-Fos pathway as “danger response” to alert the host that colonizing yeast have begun to transition to virulent hyphae [29]. Following the epithelial response to fungal infection, epithelial and immune cells with PRRs (e.g., TLRs and C-type lectin receptors (CLRs)) initiate the host immune defense cascade against the invading fungal pathogens [30]. Of the TLRs, TLR2 and TLR4 play important roles in the pathogenesis of candidiasis, such that they induce proinflammatory responses in a synergistic manner with specific CLRs [30,31]. A previous investigation involving an in vivo oropharyngeal candidiasis mouse model revealed that TLR2 mRNA expression in oral tissues is strongly induced by *C. albicans* in a sustained manner for up to 72 h after infection, whereas the TLR4 mRNA expression in oral tissues peaks at 6 h and then gradually declines until 48 h after infection [32]. Among the CLRs, Dectin-1 and Dectin-2 are the major sensors that participate in *C. albicans* recognition and the subsequent induction of anti-fungal signaling pathways [30,31]. Expression of these CLRs is immediately induced within the 24 h after *C. albicans* infection [33]. These findings are consistent with our results, whereby distinct temporal expression patterns were observed with respect to PRRs induced by *C. albicans*. Phagocytic cells (e.g., neutrophils, macrophages, and dendritic cells) are important components of the immunological response to oral candidiasis. Notably, neutrophils are essential for controlling and eliminating fungal infection; they can kill *C. albicans* through both intracellular and extracellular mechanisms. Similarly, macrophage activation leads to the release of several key cytokines that are important for host protection against *C. albicans* infection. Previous analyses of the oral mucosa in mice revealed the induction of chemokines that participate in neutrophil recruitment (e.g., CXCL1) and macrophage chemotaxis (e.g., CCL2) after oral *C. albicans* infection [30,33,34,35]. Consistent with these results, we observed changes in PRR and chemokine expression levels, suggesting that *L. rhamnosus* L8020 prevented and/or reduced the *C. albicans*-related induction of PRR and chemokine gene expression during the experimental period. Notably, few genetic changes were caused by the presence of *L. rhamnosus* L8020 in oral tissues. Although in vitro microarray results using mouse gingival epithelial cells showed that more than 400 changes in gene expression occurred within 1 h of co-culture with *L. rhamnosus* L8020, only 60 changes remained after 4 h of co-culture [14]. These results imply that at 72 h after treatment with *L. rhamnosus* L8020, alterations in gene expression had already occurred and a stable state had been achieved in oral tissues.

## 5. Conclusions

In summary, we have demonstrated the in vivo efficacy of *L. rhamnosus* L8020 in a mouse model of oral candidiasis. Specifically, *L. rhamnosus* L8020 abolished the pseudomembranous region of the mouse tongue following infection by *C. albicans*; this tissue alteration was accompanied by the suppression of changes in PRR and chemokine gene expression levels. Our results suggest that *L. rhamnosus* L8020 has protective or therapeutic potential with respect to oral candidiasis, which supports its use as a potential probiotic for improved oral health management. Further proof of concept studies should be conducted in humans to verify the mechanism by which *L. rhamnosus* L8020 acts as a probiotic, thereby providing robust evidence to support its use in clinical applications.

## 6. Patents

Prof. Hiroki Nikawa has patents for *L. rhamnosus* L8020.

## Data Availability

Not applicable.

## Supplementary Materials

The following is available online at https://www.mdpi.com/article/10.3390/jof7050322/s1, Table S1: Primers and probes used for real-time PCR.
