# Supplementary material for: In Vivo Efficacy of Lacticaseibacillus rhamnosus L8020 in a Mouse Model of Oral Candidiasis
Source: J Fungi (Basel). 2021 Apr 21;7(5):322. doi: 10.3390/jof7050322 (PMC8143095; doi:10.3390/jof7050322)
Supplement: Supplementary file 1 [file jof-07-00322-s001.zip › Ito_et_al_SupTable1.pdf]

Table S1. Primers and probes used for real-time PCR.

Target name		Sequence (5'–3')
CCL2	Primer F	ATGCAGGTCCCTGTCATGC
	Primer R	CATCTTGCTGGTGAATGAGTAGC
	Probe	6FAM-TCTGGGCCTGCTGTTCACAGTTGC-TAMRA
CXCL1/KC	Primer F	CTGCACCCAAACCGAAGTC
	Primer R	AGCTTCAGGGTCAAGGCAAG
	Probe	6FAM-CACTCAAGAATGGTCGCGAGGC-TAMRA
Dectin-1	Primer F	ACAAGCCCACAGAATCATCTTTAG
	Primer R	GATCCAATTAGGAAGGCAAGACTG
	Probe	6FAM-AGGTGGCTCCCTCCAAGGCATCCC-TAMRA
Dectin-2	Primer F	AACCCCTGACCTTCTGAACATAC
	Primer R	CGCAATGAAACAGGTACTCAAGAG
	Probe	6FAM-ATCACAGCAGCTGACCAGAGTCTCAGG-TAMRA
TLR-2	Primer F	GGAACTGTCGGAGGTAGAGTTCG
	Primer R	TTTCTACTTTACCCAGCTCGCTCA
	Probe	6FAM-ACCCTCAATGGGCTCGGCGATTTC-TAMRA
TLR-4	Primer F	AAACTTGCCTTCAAAACCTGGC
	Primer R	ACCTGAACTCATCAATGGTCACATC
	Probe	6FAM-CACGTCCATCGGTTGATCTTGGGAGAA-TAMRA
β-actin	Primer F	CCACACTGTGCCCATCTACG
	Primer R	GTGGTGGTGAAGCTGTAGCC
	Probe	6HEX-CCTGCGTCTGGACCTGGCTGGC-TAMRA

Note: Forward primers (Primer F), reverse primers (Primer R), and probes are listed.

Table. S1
